# The exploration of miRNAs and mRNA profiles revealed the molecular mechanisms of cattle-yak male infertility

**DOI:** 10.3389/fvets.2022.974703

**Published:** 2022-10-05

**Authors:** Shaokang Zhao, Wenqiang Sun, Shi-Yi Chen, Yuchao Li, Jie Wang, Songjia Lai, Xianbo Jia

**Affiliations:** Farm Animal Genetic Resources Exploration and Innovation Key Laboratory of Sichuan Province, Sichuan Agricultural University, Chengdu, China

**Keywords:** cattle-yak, miRNA, mRNA, bta-miR-7, male sterility

## Abstract

Cattle-yak, the first-generation offspring of cattle and yak, inherited many excellent characteristics from their parents. However, F1 male hybrid infertility restricts the utilization of heterosis greatly. In this study, we first compared the testicular tissue histological characteristics of three cattle, three yaks, and three cattle-yak. Then we explored the miRNA profiles and the target functions of nine samples with RNA-seq technology. We further analyzed the function of DE gene sets of mRNA profiles identified previously with GSEA. Testicular histology indicated that the seminiferous tubules became vacuolated and few active germ cells can be seen. RNA-seq results showed 47 up-regulated and 34 down-regulated, 16 up-regulated and 21 down-regulated miRNAs in cattle and yaks compared with cattle-yak, respectively. From the intersection of DE miRNAs, we identified that bta-miR-7 in cattle-yak is down-regulated. Target prediction indicated that the filtered genes especially MYRFL, FANCA, INSL3, USP9X, and SHF of bta-miR-7 may play crucial roles in the reproductive process. With further network analysis and GSEA, we screened such hub genes and function terms, we also found some DE gene sets that enriched in ATP binding, DNA binding, and reproduction processes. We concluded that bta-miR-7 may play an important role in influencing fecundity. Our study provides new insights for explaining the molecular mechanism of cattle-yak infertility.

## Introduction

Studies have revealed that only about 2% of genome transcripts have protein-coding ability (1). However, as the most numerous components of genomic transcriptional products, the non-coding RNAs have also been confirmed that play significant roles in regulating gene expression at different levels such as transcription, RNA processing, translation, and epigenetic regulation (2–4). The non-coding RNAs can be classified into small and long ones based on length (5). microRNAs (miRNAs) are endogenous small non-coding RNA molecules 19–25 nucleotides in size that regulate posttranscriptional silencing of target genes, and single miRNAs can influence the expression patterns of many genes by targeting hundreds of mRNAs (6). In mammals, many studies have focused on miRNA and mRNA and increasing evidence had demonstrated that miRNAs can participate in multiple biological processes such as proliferation (7), differentiation (8, 9), and apoptosis (10). Moreover, miRNAs can act as epigenetic modulators to affect the protein levels of the target mRNAs without modifying the gene sequences, and the expression of miRNAs is also regulated by epigenetic machinery such as DNA methylation, and RNA modification (11). In recent years, there have also been a large number of related studies on the regulation of azoospermia and male sterility by miRNAs.

Cattle-yak, an excellent hybrid of cattle (*Bos taurus*) and yak (*Bos grunniens*), exhibits better economic traits than its parents such as meat quality, cold resistance, and growth performance. For example, the phospholipids containing long-chain polyunsaturated fatty acids have been confirmed more abundant in the muscles of plateau cattle than plain cattle, and are considered beneficial to human health (12–14). But the Haldane's rule demonstrates that the heterogametic sex of first-generation hybrids is infertile. Male infertility of F1 generation of cattle-yak resulting from spermatogenic arrest has greatly restricted the effective utilization of heterosis from the hybrids and the cultivation of new breeds (15, 16). Male cattle-yak have normal external genitalia, libido, and mating behavior, but testicular histology revealed that its seminiferous tubule walls were thinner and spermatogenesis was arrested at the stage of spermatogonia differentiation, which was hardly ever able to proceed further than meiosis I (17, 18). In epigenetics, unlike yaks, the expression levels of acetyl-histone H3 Lys9 (*ACK9*) in cattle-yak were abnormal and the 5-methylcytosine level was higher (19). Studies also observed that the androgens levels were decreased and androgen receptors led to higher expression of 3β-hydroxysteroid dehydrogenase(*3*β*HSD*) in the Leydig cells of cattle-yak testes (20). As a member of deleted azoospermia family genes, the *Boule* gene has also been proven to play important role in regulating primordial germ-cell formation (21). Entirely different from cattle, the expression level of the *Boule* gene was lower because the long CpG island in the promoter was hypermethylated in the testes of cattle-yak hybrids, and the methylation level was higher in the core promoter than outside (22). With the development of molecular biology in recent decades, such genes and transcription factors associated with male infertility had been identified. For example, DNA-binding protein *PRDM9* is involved in double-strand breaks initiating meiotic recombination, and the position of the double-strand breaks (DSBs) is closely related to the fertility of male hybrids (23). Researchers had found that the expression level of bovine meiosis defective 1 (*bMei1*) participated in spermatogenesis was significantly decreased and the methylation level of promoter and gene body was significantly increased in the testis of cattle–yak compared with cattle. On the contrary, with the treatment of methyltransferase inhibitor 5-aza-2′ deoxycytidine (*5-Aza-CdR*), the activation of *bMei1* would recover, further illustrating the association between *bMei1* expression level and methylation (24). Besides, as a subfamily of the Argonaute protein, PIWI-like protein 1 (*PIWIL1*) plays a crucial role during germ-line development, stem cell maintenance, spermatogenesis, and transposon regulation, and the expression levels of *PIWIL1* mRNA and *PIWI* protein in the testes of cattle-yak are significantly lower than that of cattle or yaks because of hypermethylation (25–29). In addition, *Rad51* and meiotic-specific *DMC*1 recombinases can act synergistically in the process of homologous recombination that is important for spermatogenesis. A previous study had proved the levels of *Rad51* and *DMC1* are extremely decreased in the male cattle-yak testis with a corresponding higher incidence of germ cell apoptosis (30). As a family of transcription factors, heat shock factors can regulate the expression levels of heat shock proteins (*HSP*) and perform crucial roles during gametogenesis and development in physiological conditions, infertility of cattle-yak may be caused by the upregulation of *HSP53* (31–33). With the development of high-throughput sequencing technology, many studies had preliminary performed comparative transcriptome profiles analysis in testes tissues and epididymis of cattle-yak and yak (34–37). However, few studies explored the miRNA profiles and the function of their target genes of cattle, yak, and cattle-yak comprehensively.

In our previous study, we compared the lncRNA and mRNA profiles of the testicular tissues for the differential expression analysis of cattle, yaks, and cattle-yak (38). In this study, we also used RNA-seq technology to conduct the comparison of three varieties of miRNAs profiles and their target genes. we also performed GSEA of DE gene sets and network analysis to identify the differences in gene expression patterns. We hope to find some significant biological markers and function terms associated with cattle-yak male sterility and have a deeper understanding of the molecular mechanism of this problem.

## Results

### Testicular tissue histological characteristics of nine samples

**Figure 1:**
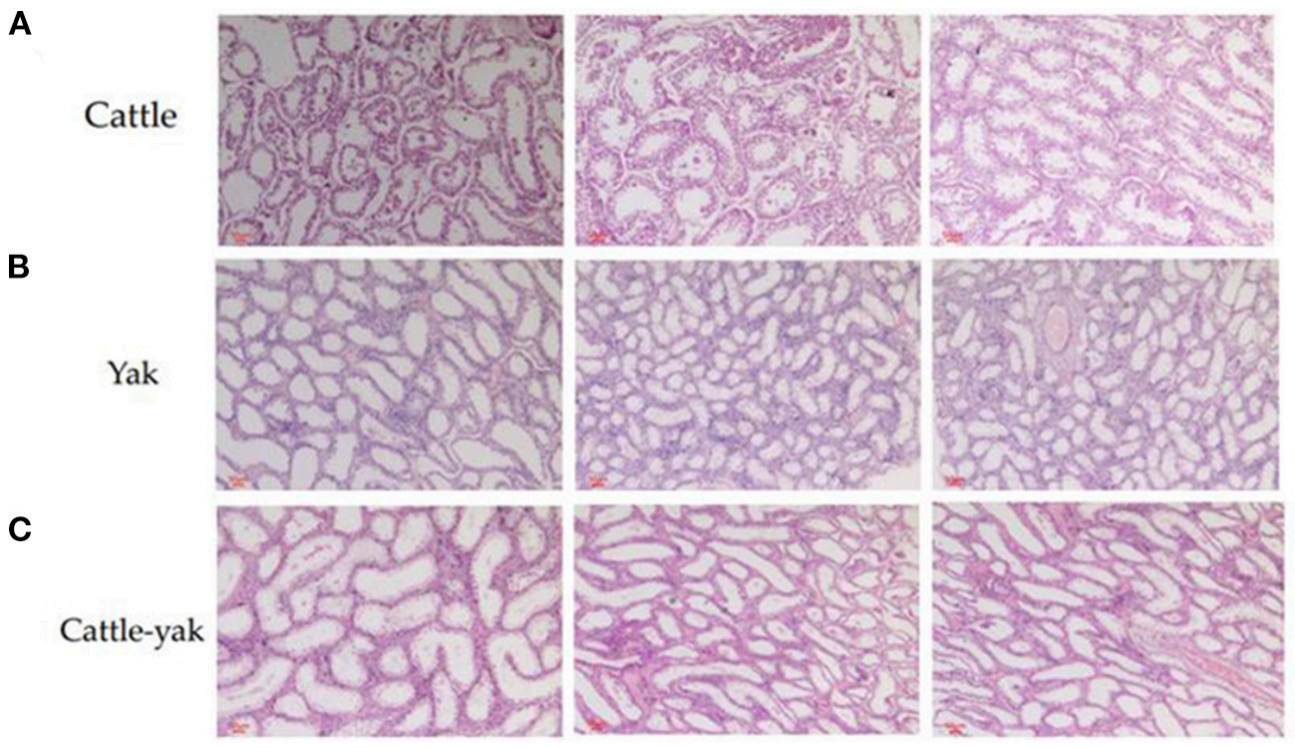
The histological differences among the testis of cattle, yak, and cattle-yak. The cattle longitudinal section of testicular tissues **(A)** and yak longitudinal section of testicular tissues **(B)** showed that the relatively thick seminiferous tubules filled with spermatogonia, spermatocytes, and spermatids. On the contrary, seminiferous tubules of cattle-yak **(C)** were highly vacuolated and there are few spermatids can be seen.

Compared with cattle and yaks, the testicular tissue sections of cattle-yak showed that seminiferous tubules were highly vacuolated and the walls became thinner. The reverse of cattle and yak (Figures 1A,B), the area of the longitudinal section of the testis was expanded and there are few germ cells or spermatocytes can be seen in the seminiferous tubules of cattle-yak (Figure 1C).

### Quality control of sequencing data

After removing the 3' adaptors with cutadapt and filtering the low-quality reads with trimmomatic, clean reads were finally converted to FASTA format. There were 3,192,697–6,617,422 unique clean reads generated by filtering low-quality and covering the repeated reads. The GC percentage of these reads was from 46.31 to 49.29%, and all the Q30 values of these reads were surpass 96% (Table 1).

**Table 1:** The statistics results of clean data.

**Sample**	**Reads count**	**Unique count**	**Bases count**	**Average length**	**Q20**	**Q30**	**GC percentage**
H1	12,855,276	3,351,413	360,561,690	28.05	97.87%	96.54%	47.02%
H2	12,886,450	3,783,343	367,250,682	28.5	97.78%	96.38%	46.35%
H3	15,070,070	4,342,944	424,855,422	28.19	97.76%	96.34%	46.31%
M1	10,694,999	3,881,169	278,614,344	26.05	97.78%	96.40%	48.41%
M2	12,384,809	4,380,294	331,397,620	26.76	97.68%	96.22%	47.51%
M3	14,831,950	5,470,038	382,097,489	25.76	97.72%	96.32%	47.80%
P1	13,443,245	4,640,237	347,901,405	25.88	97.84%	96.52%	48.62%
P2	14,979,305	6,617,422	392,403,979	26.2	97.80%	96.44%	48.67%
P3	12,329,720	3,192,697	317,680,881	25.77	97.82%	96.47%	49.29%

### Reads mapping and filtration

Blastn was used to align the reads to the Rfam database (rRNA, snRNA, snoRNA, tRNA) and then filtered the mapped reads with gapopen =0, evalue <0.01, and mismatch ≤ 1 (Supplementary Table 1). Bowtie was used to map the exon and intron sequence with mismatch ≤ 1 and then filtered the reads that can be mapped to the exon sequence and can not be mapped to the intron sequence. Clean reads were mapped to the bovine reference genome (ARS-UCD1.3) with mismatch ≤ 1 (Table 2).

**Table 2:** The result of reads mapped to the reference genome.

**Sample**	**Database**	**Total_reads**	**Total_percentage**	**Unique_reads**	**Unique_percentage**
h1	dna	5448647	0.423845	1772587	0.528907
h2	dna	6139838	0.476457	2086695	0.551548
h3	dna	7122441	0.472622	2373291	0.546471
m1	dna	4476534	0.418563	1954724	0.503643
m2	dna	5736035	0.463151	2359750	0.53872
m3	dna	6308983	0.425364	2757348	0.504082
p1	dna	5515553	0.410284	2342296	0.504779
p2	dna	6074855	0.40555	3236625	0.489107
p3	dna	4997417	0.405315	1595278	0.499665

### The miRNAs family and expression level analysis

**Figure 2:**
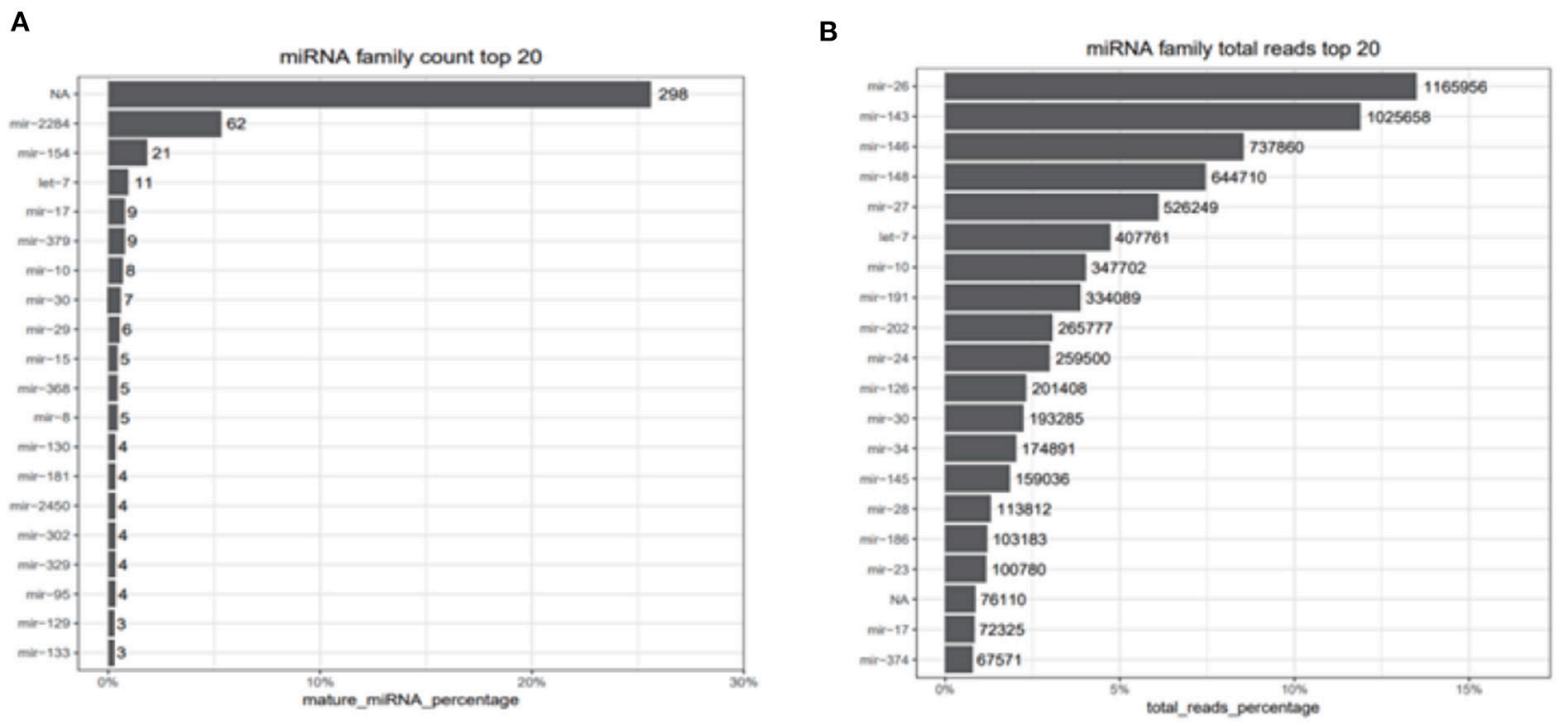
miRNAs family count top 20 and total reads top reads. **(A)** Top 20 counts of mature miRNAs from one miRNAs family. **(B)** Top 20 counts of reads of these miRNAs. NA represents that the reads are consistent with the characteristics of miRNAs but didn't belong to any miRNA families we identified.

**Figure 3:**
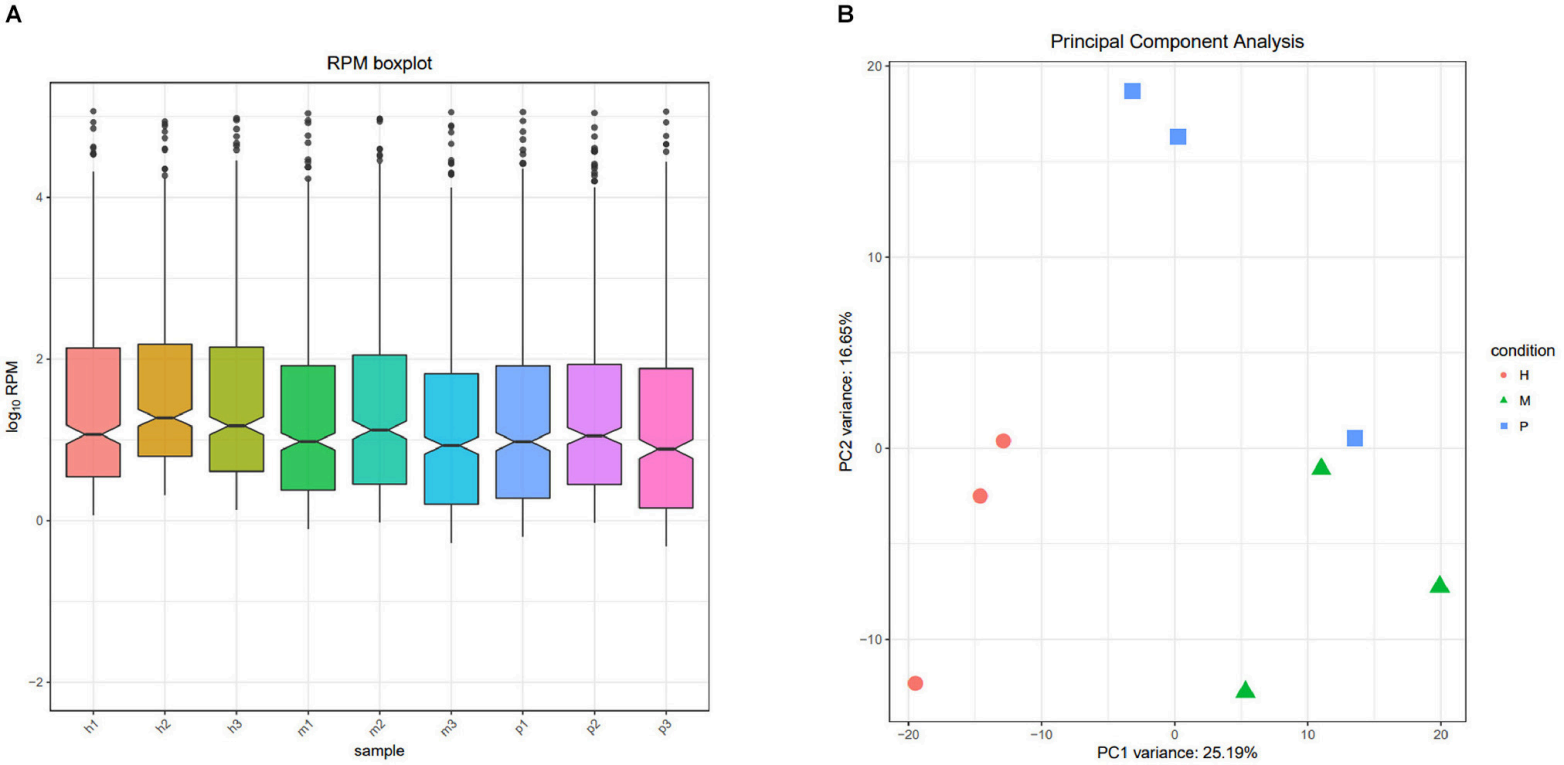
The RPM distribution and principal component of nine samples. **(A)** The RPM boxplot of nine samples, the *x-*axis represents each sample, and the *y-*axis represents log_10_ RPM. **(B)** Principal component analysis of nine samples, the *x-*axis represents the PCA1, and the *y-*axis represents the new dimension PCA2, each color and shape represents each variety.

**Figure 4:**
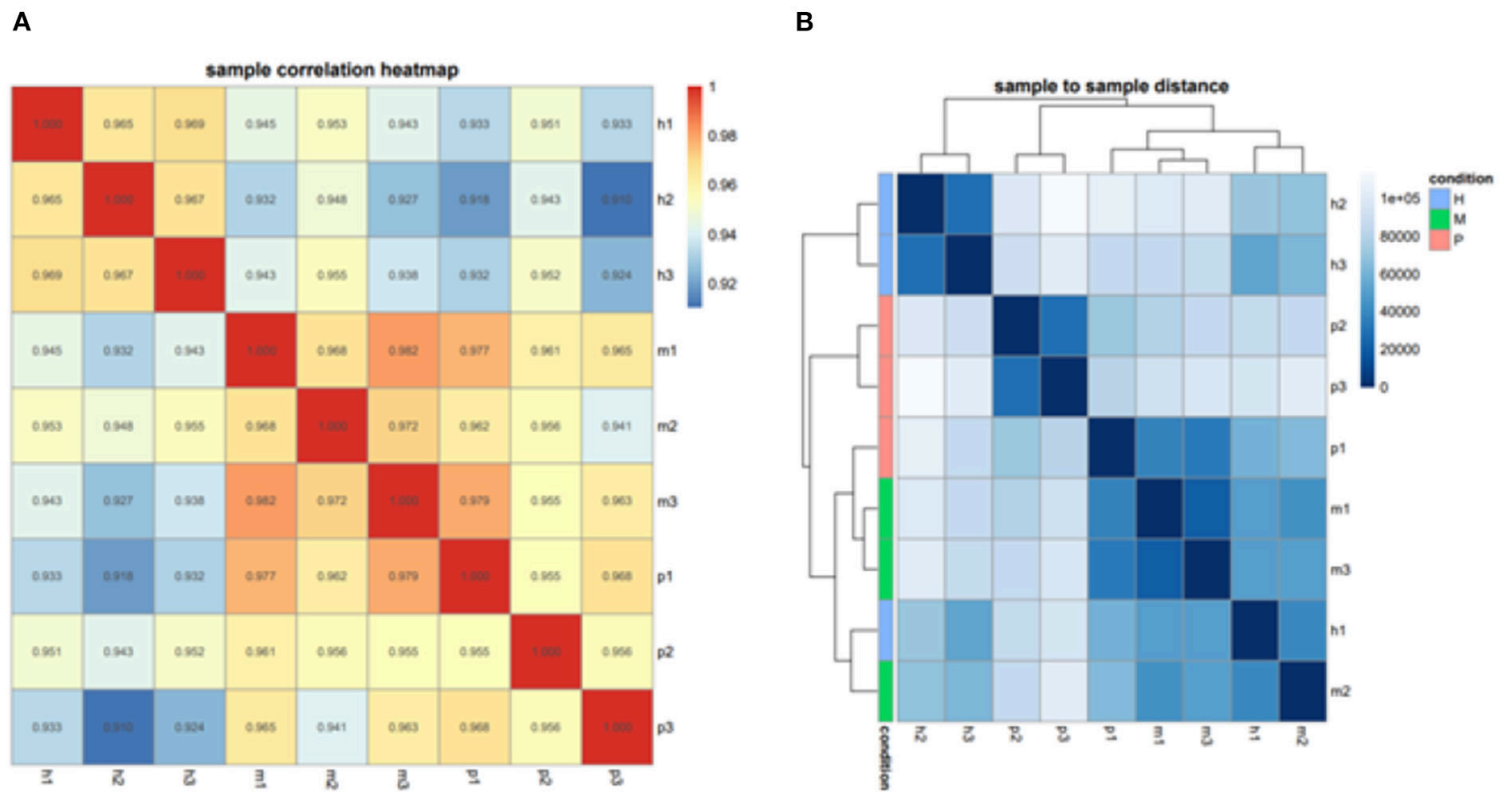
The correlation and distance among nine samples. **(A)** Sample correlation heatmap of nine samples, each small square represents the correlation between each sample of horizontal and vertical coordinates. The larger the correlation coefficient, the closer the color is to red. **(B)** Sample to sample distance heatmap of nine samples, each small square represents the distance between each sample of horizontal and vertical coordinates. The farther the samples are, the lighter the color.

After filtering the reads that did not belong to miRNAs, the mirDeep2 was used to predict the novel miRNAs and secondary structure of miRNAs. After quantification of miRNAs, we analyzed the miRNAs family to further understand the function of miRNAs to their target genes (Figure 2). Then these counts were normalized with RPM. The RPM distribution of nine samples can be seen as the RPM boxplot (Figure 3A). At the same time, we analyzed the principal component of nine samples. These nine samples can be divided into H, M, and P groups (Figure 3B). With the miRNAs expression levels of each sample, we further analyzed the correlation between three samples in one group and the distance among three groups, the samples of each group were highly relevant and the distance among three groups was obvious (Figure 4).

### Differential expression analysis of miRNAs among nine samples

**Figure 5:**
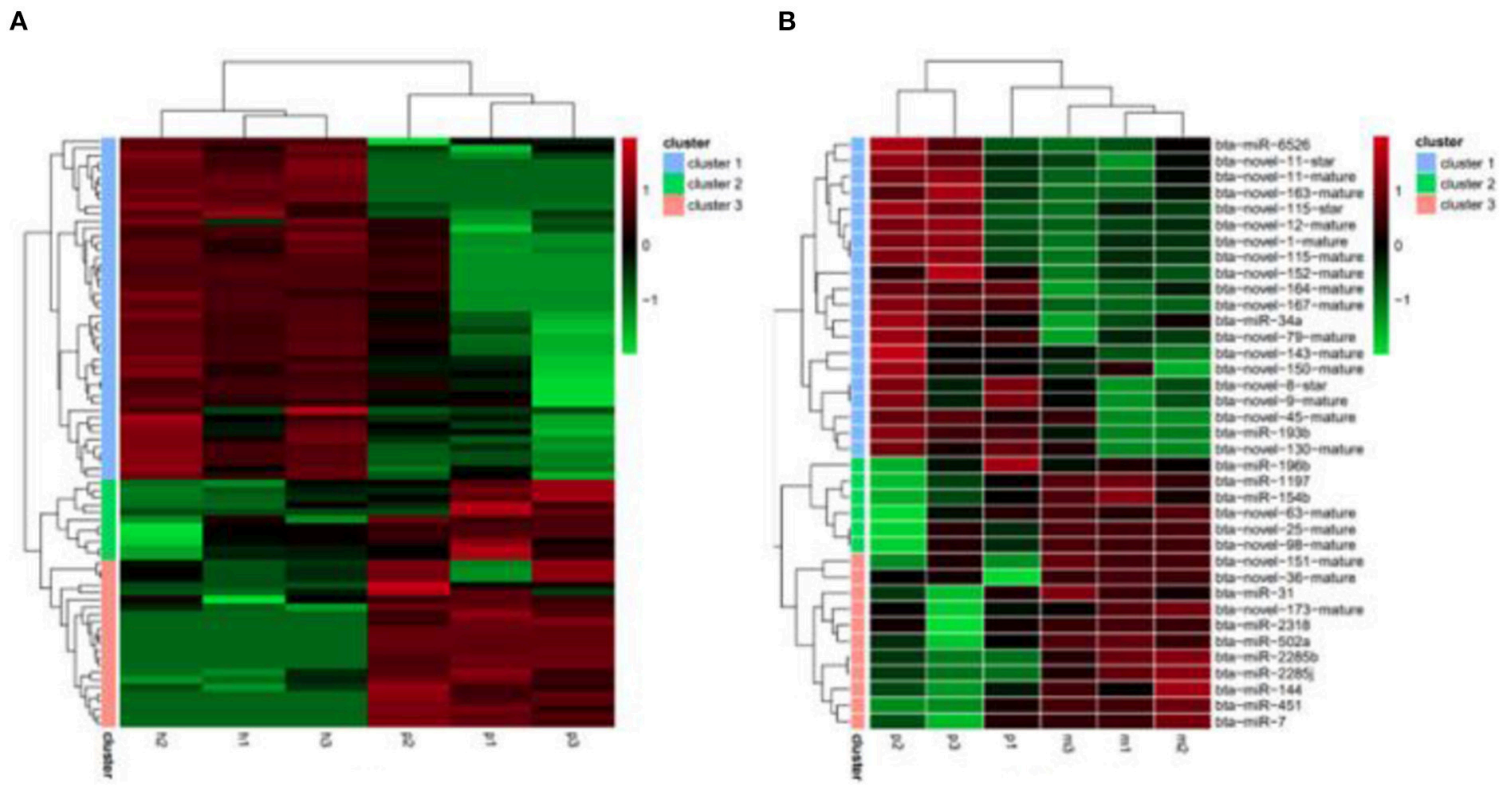
Heatmap of clustering analysis and differentially expression of H vs. P and M vs. P. **(A)** Heatmap of differentially expression analysis of H vs. P. **(B)** Heatmap of differentially expression analysis of M vs. P. Red represents the high expression of miRNA of each comparison group, and green represents the low ones.

**Figure 6:**
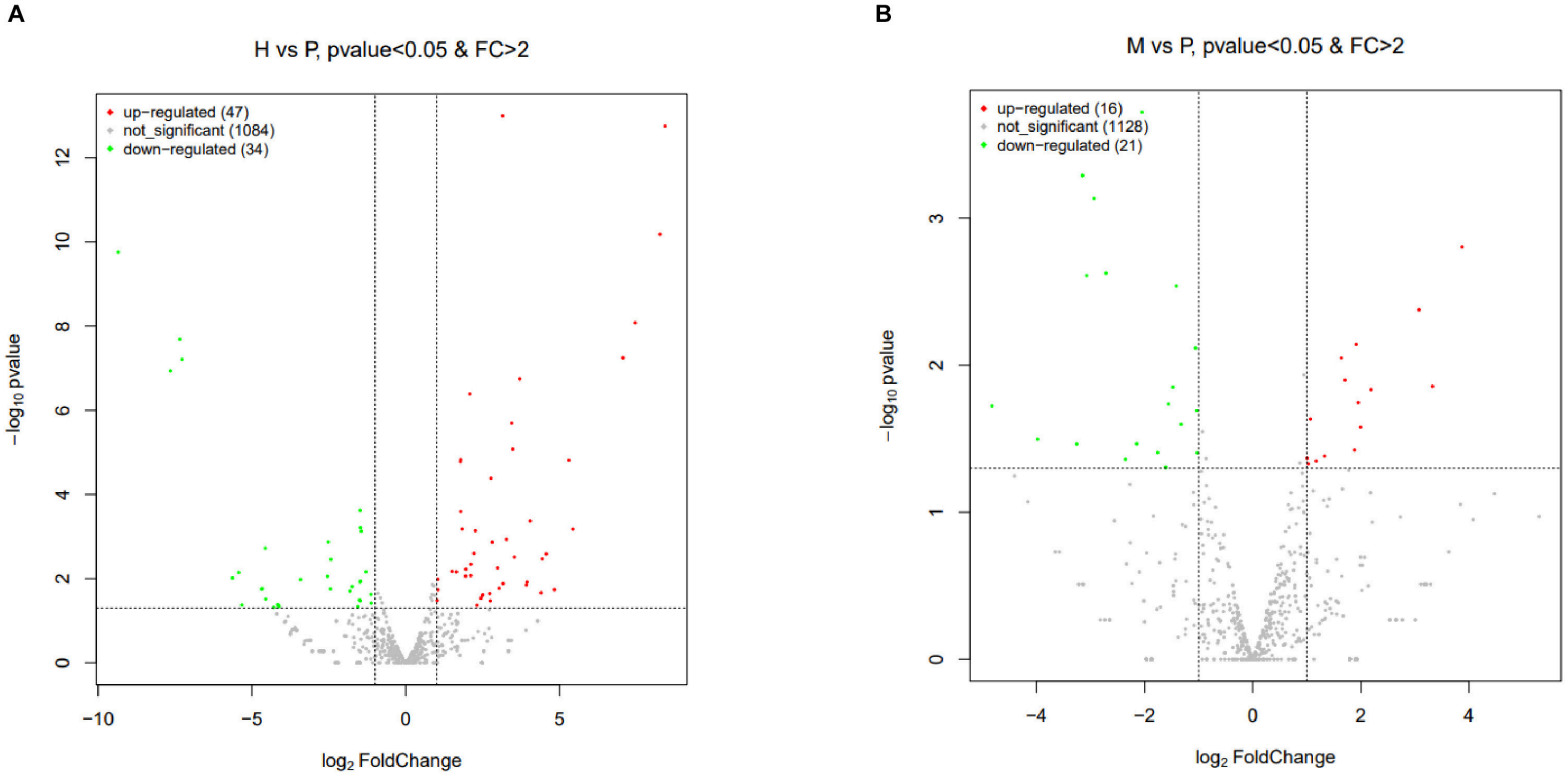
Volcano plots of differentially expressed miRNAs of H vs. P and M vs. P. **(A)** Volcano plot of differentially expressed miRNAs between cattle (H) and cattle-yak (P). **(B)** Volcano plot of differentially expressed miRNAs between yak (M) and cattle-yak (P). Each point represents each DE miRNA, red represents u*p-*regulated and green represents down-regulated.

**Figure 7:**
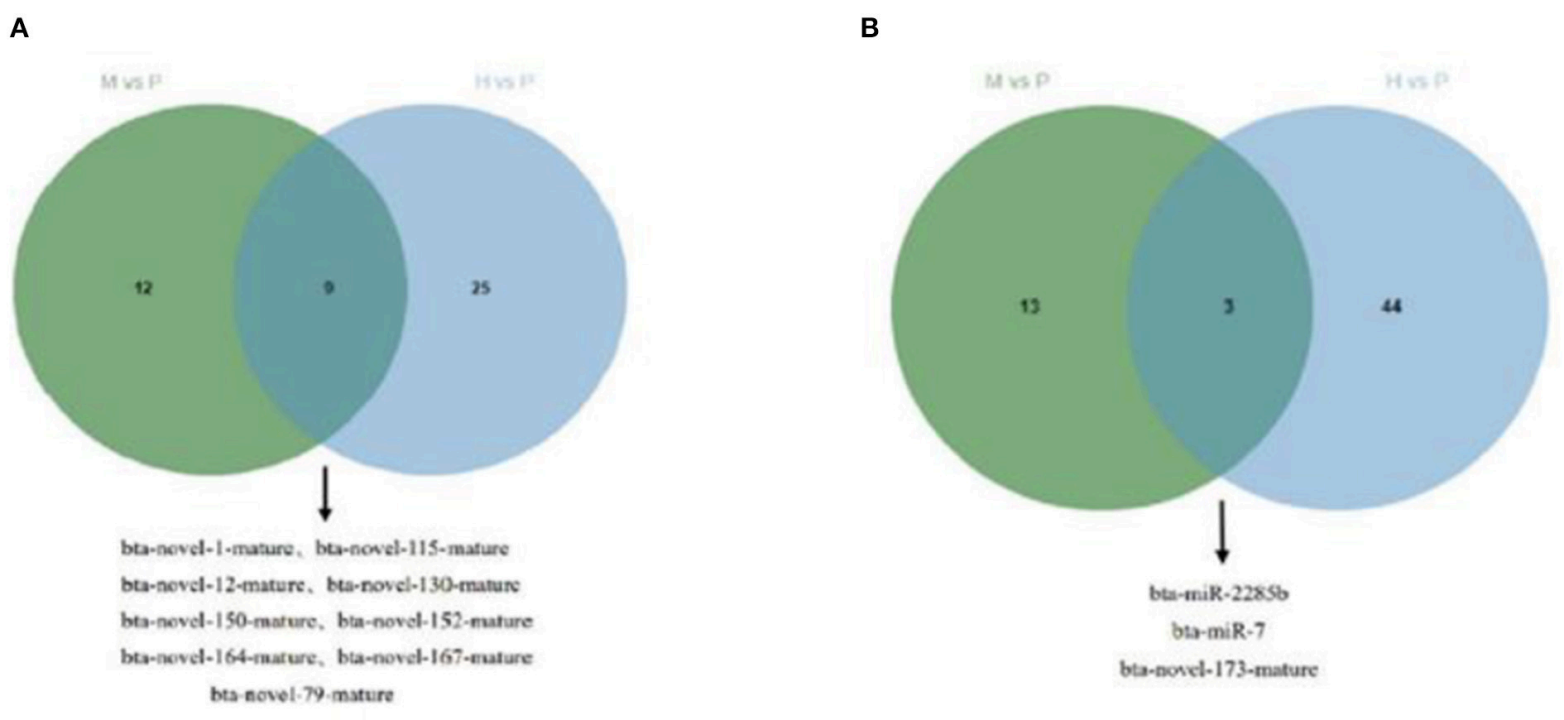
Venn plot of cattle-yak miRNAs in the merged analysis between H vs. P and M vs. P. **(A)** Venn plot of u*p-*regulated miRNAs of cattle-yak in the merged analysis between H vs. P and M vs. P. **(B)** Venn plot of down-regulated miRNAs of cattle-yak in the merged analysis between H vs. P and M vs. P.

The R package edgeR was used to analyze the differential miRNAs expression among nine samples with CPM >1, *p-*value < 0.05, |log_2_FoldChange| >1. And then the clustering analysis and differentially expression of H vs. P and M vs. P were then diagramed with a heatmap (Figure 5). There were 81(47 up-regulated and 34 down-regulated) and 37(16 up-regulated and 21 down-regulated) differentially expressed miRNAs in H vs. P and M vs. P, respectively (Figure 6). The list of differentially expressed miRNAs can be seen in Supplementary Table 2. Then, we consolidate the common up-regulated and down-regulated miRNAs of cattle-yak in the merged analysis of H vs. P and M vs. P (Figure 7).

### Target prediction, functional enrichment, and network analysis

With the list of the differentially expressed miRNAs of H vs. P and M vs. P, we used Miranda software to predict their target genes. In the H vs. P group, there were 6 up-regulated and 1 down-regulated miRNA in cattle that can target prediction successfully (Table 3).

**Table 3:** The list of differentially expressed miRNAs and their target genes of H vs. P.

**MiRNAs id**	**Regulated**	**Target genes**
bta-miR-122	Up	HFE, CD151, SEMA4D, ADAMTS14,
bta-miR-146a	Up	YTHDF2
bta-miR-184	Up	EIF2S2, C7H5orf15, STOML2, FGFR1OP,
bta-miR-34c	Up	ESPL1, KDM6A, THBS2, BCORL1, ZNF568, ALKAL2,
bta-miR-7	Up	MYRFL, FANCA, INSL3, USP9X, SHF
bta-miR-93	Up	SUFU, LOC789812, LOC789787, RALGAPA1, SEPHS1, NETO2, TMEM187, USP32, STOX2, RPAP2
bta-miR-455-3p	Down	SMAD2, CAMKV, LDAH, GREB1, ANKS6, XRN1, CLEC7A, PIK3R1, PHF7, LOC787074, SPHK2, LINS1, MKRN3, IFIT3, RPAP2, TRPM2, ZNF217

**Figure 8:**
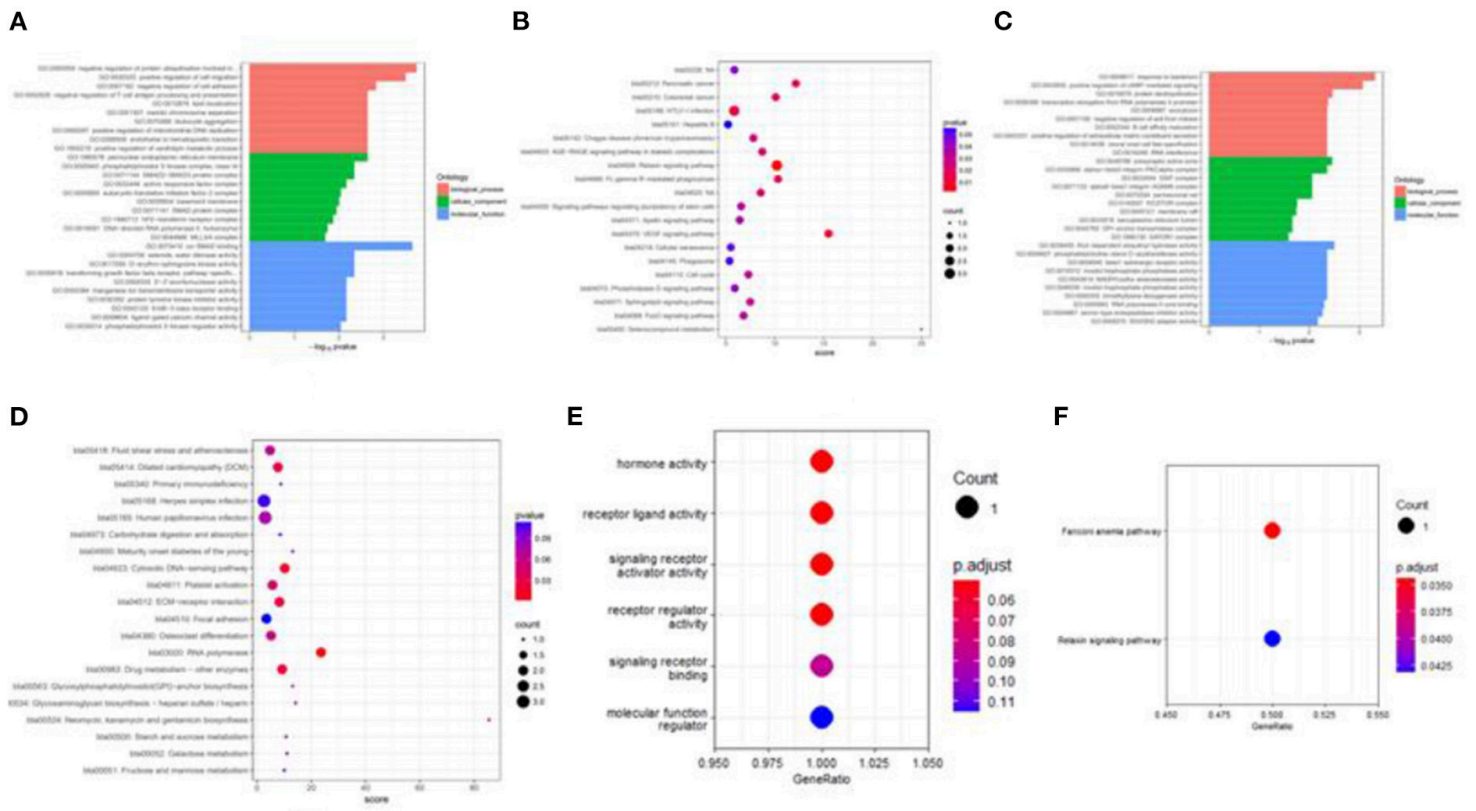
Target genes GO and KEGG enrichment top bar plot and point plot of different groups. **(A)** Top 30 bar plot of enriched GO terms of DE miRNAs target genes in the H vs. P group. **(B)** Top 20 points plot of KEGG pathway of DE miRNAs target genes in the H vs. P group. **(C)** Top 30 bar plot of enriched GO terms of DE miRNAs target genes in the M vs. P group. **(D)** Top 20 points plot of KEGG pathway of DE miRNAs target genes in the M vs. P group. **(E)** Point plot of miR-7 target genes enriched GO terms. **(F)** Point plot of miR-7 target genes enriched pathways.

**Figure 9:**
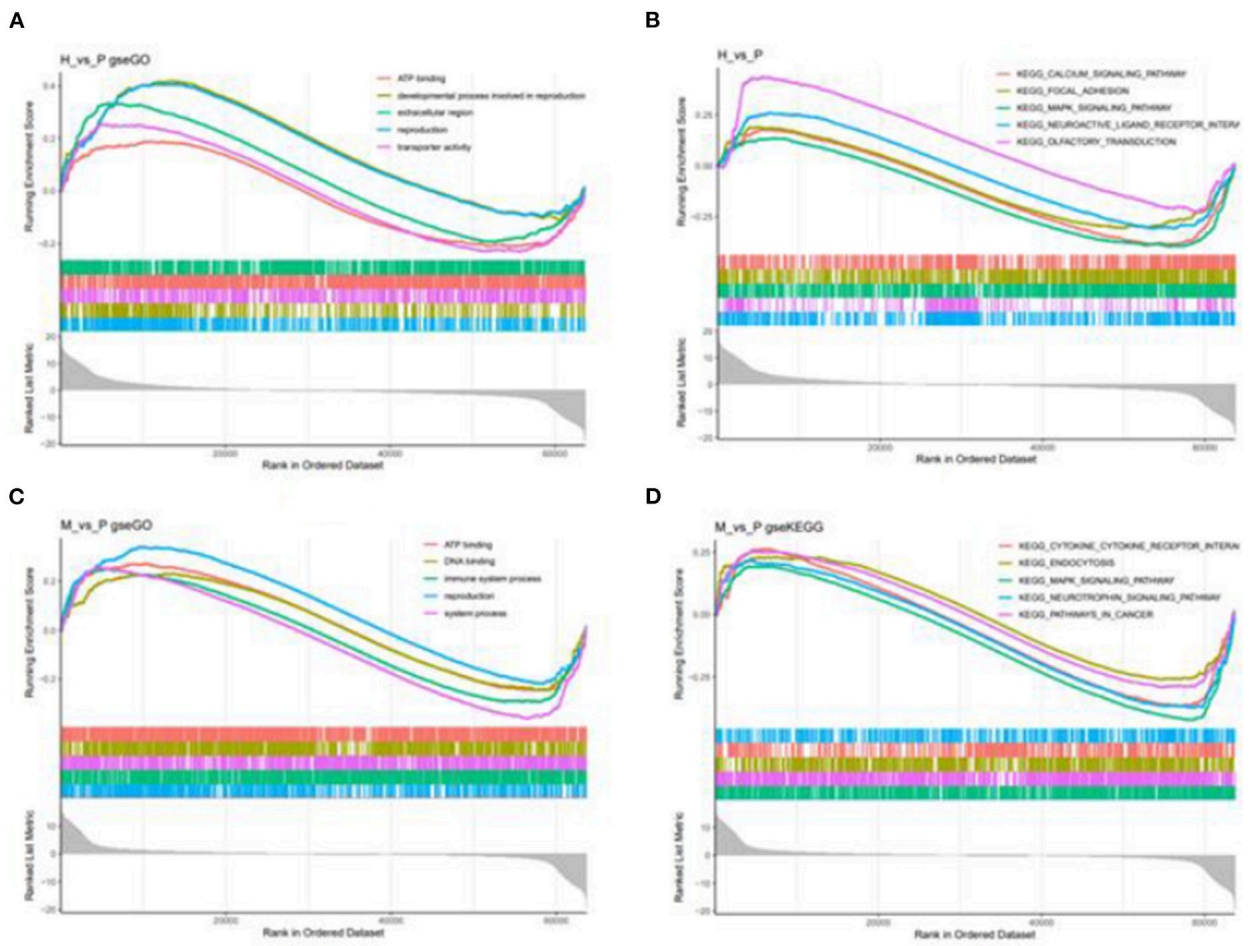
GSEA enrichment plot of differentially expressed genes in H vs. P and M vs. P. **(A)** The first five go terms of DEG in the GSEA-GO analysis of H vs. P. **(B)** The first five KEGG pathways of EDG in the GSE-KEGG analysis of H vs. P. **(C)** The first five go terms of DEG in the GSE-GO analysis of M vs. P. **(D)** The first five KEGG pathways of EDG in the GSEA-KEGG analysis of M vs. P. One curve for each color represents each term or pathway.

**Figure 10:**
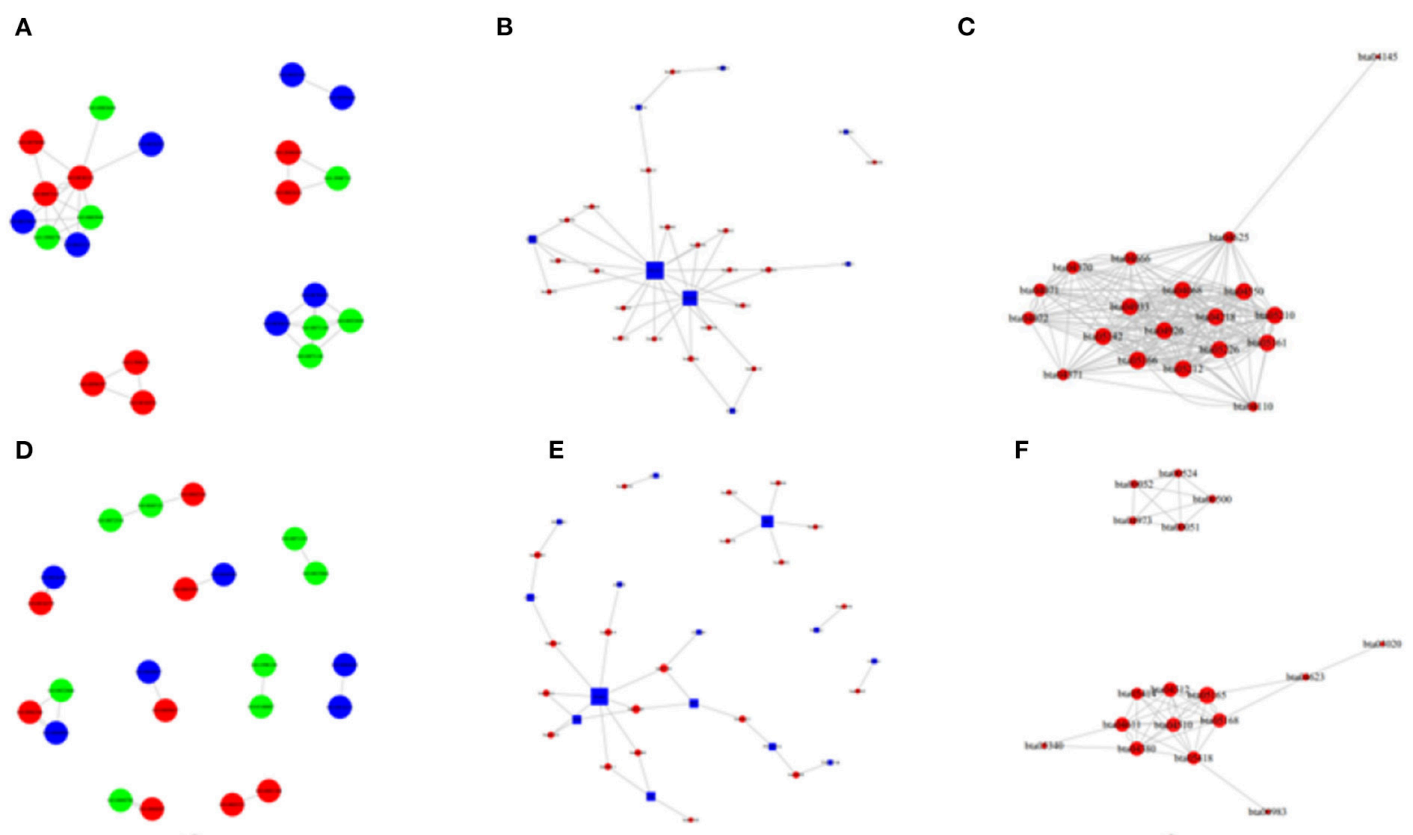
The network of GO enriched terms and pathways in the H vs. P and M vs. P groups. **(A)** The GO to GO network of u*p-*regulated terms in cattle between the comparison of cattle and cattle-yak. **(B)** The pathway enrichment top network between the comparison of cattle and yak. **(C)** The pathway to pathway top network between of u*p-*regulated in cattle between he comparison of cattle and cattle-yak. **(D)** The GO to GO network of u*p-*regulated terms in yak between the comparison of yak and cattle-yak. **(E)** The pathway enrichment top network between the comparison of yak and cattle-yak. **(F)** The pathway to pathway top network between of u*p-*regulated in yak between he comparison of yak and cattle-yak.

Besides, M vs. P group filtered 2 up-regulated and 3 down-regulated miRNAs in yak can target the protein-coding genes and further regulate the associated signaling pathways (Table 4). We also observed that bta-miR-7 is commonly down-regulated in cattle-yak between H vs. P and M vs. P, the predicted target genes MYRFL, FANCA, INSL3, USP9X, and SHF may play significant roles in cattle-yak male-sterility. With the predicted genes, we carried out GO and KEGG enrichment analyses to annotate their function (Supplementary Tables 3–6). We selected the top 10 GO terms of molecular function, biological process, cellular component in GO enrichment, and the up-regulated genes of cattle most engaged in the negative regulation of ubiquitin-dependent protein catabolic process (GO:2000059), positive regulation of cell migration (GO:0030335), thiol–dependent ubiquitinyl hydrolase activity (GO:0036459), and positive regulation of mitochondrial DNA replication (GO:0090297). Besides, KEGG enrichment analysis demonstrated that these target genes are most enriched in the cell cycle (bta04110), RNA transport(bta03013), and PI3K–Akt signaling pathway(bta04151), Focal adhesion (bta04510) pathways. On the other hand, the up-regulated genes in yak are most enriched in the DNA-binding transcription factor activity (GO:0003700), positive regulation of DNA demethylation (GO:1901537), protein deubiquitination (GO:0016579), zinc ion binding (GO:0008270) terms and relaxin signaling pathway (bta04926), RNA polymerase (bta03020) pathways, such the up-regulated GO terms and pathways in cattle and yak may positively participate in the reproductive processes (Figures 8A–D). With the analysis of miR-7 target genes function, we found these genes enriched in hormone activity, signaling receptor binding and activator activity, molecular function regulator process, Fanconi anemia pathway, and relaxing signaling pathway that is associated with hormone secretion and signal transduction (Figures 8E,F). At the same time, we performed Gene Set Enrichment Analysis (GSEA) to interpret the enrichment results of DEG sets. We found that the gene sets of cattle enriched in the ATP binding, reproduction, and developmental process involved in reproduction processes and focal adhesion, olfactory transduction, and MAPK signaling pathway in the H vs. P group. Likewise, the gene sets of yaks are most involved in the ATP binding, DNA binding, and reproduction processes, as well as the cytokine receptor interaction and MAPK signaling pathways (Figure 9). We also found that the positive regulation of cell migration (GO:0030335), negative regulation of cell adhesion (GO:0007162), SMAD2-SMAD3 protein complex processes, PIK3R1 gene, SMAD2 gene, relaxin signaling pathway (bta-04926) and cellular senescence (bta04218) were located in the center of GO and KEGG regulatory network of H vs. P DE miRNAs target genes (Figures 10A–C). Similarly, in the M vs. P DE target genes GO and KEGG regulatory network, membrane raft (GO:0045121) process, and focal adhesion (bta04510) pathways were in the central location. The ITGB3 gene may be the hub genes among these pathways (Figures 10D–F). All the enrichment results demonstrated that the male sterility of cattle-yak may be associated with a lack of regulation of such processes.

**Table 4:** The list of differentially expressed miRNAs and their target genes of M vs. P.

**MiRNAs id**	**Regulated**	**Target genes**
bta-miR-31	Up	SUPT4H1, TWISTNB, ITGB1BP2, MICAL2, USP34, NR5A2, LOC112445404, DIS3
bta-miR-7	Up	MYRFL, FANCA, INSL3, USP9X, SHF
bta-miR-193b	Down	BTK, CALU, SLC39A5, GPR61, SLC25A19, USP21, GJC2, POLR3GL, RBFOX3, SERPINB9, ANKRD54, WFIKKN2, ACBD6
bta-miR-196b	Down	POC1A, LCAT, IMPDH1
bta-miR-34a	Down	HK1, GPAA1, SLA, IRF3, EIF2B4, SERPINB8, GSTT2, TMLHE, SLC13A4, MGC133764, MRTO4, CDK18, FBH1, EXTL1, INPP5K, RIMS2, RGCC, LYPD5, ZNF771, CDC73, GSC, PRSS12, SS18L2, LRRC8E, TNR, BAHD1, ITGB3, NRDE2, NTN5, CYP2D14, ADRB1, SZT2, CCNJL, SIGLEC1, SPEN, LOC530348, RTP3, CRYBG1, TMEM229B, HSPA14, CNNM2, FAM120B, CABIN1, LOC525426, MAP4, C18H19orf84, ATRNL1, STOX2, CHADL, RIMS4

### qPCR validation

**Figure 11:**
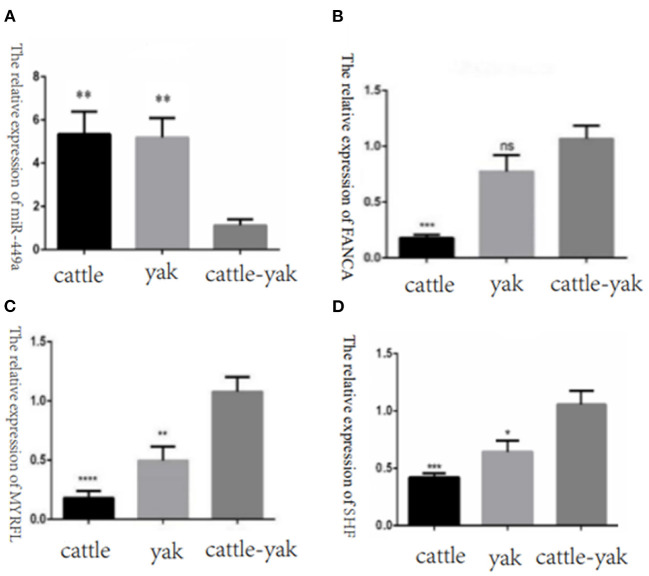
The relative expression of selected DE miRNAs and target genes in cattle, yak, and cattle-yak groups with qPCR validation. **(A)** The relative expression of miR-449a in cattle, yak, and cattle-yak. **(B)** The target gene FANCA relative expression of bta-miR-7 in cattle, yak, and cattle-yak. **(C)** The target gene MYRFL relative expression of bta-miR-7 in cattle, yak, and cattle-yak. **(D)** The target gene SHF relative expression of bta-miR-7 in cattle, yak, and cattle-yak.

To confirm the relative expression levels of DE miRNAs and their target genes were consistent with the RNA-seq results, we selected the DE miRNAs bta-miR-449a, the target genes FANCA, MYRFL, and SHF of bta-miR-7 to perform the qPCR validation. We found that the miR-449a relative expression of cattle-yak was significantly lower than cattle and yak. And the target gene expression levels of FANCA, MYRFL, and SHF were significantly up-regulated in cattle-yak, which is consistent with the lower expression of cattle-yak and negative regulation of bta-miR-7 (Figure 11).

## Discussion

As important post-transcriptional non-protein-coding regulators, miRNAs engaged in many cellular and biological processes such as proliferation, apoptosis, differentiation, and tumorigenesis. miRNAs most mediate negative post-transcriptional regulation of their targets by base pairing to sequence motifs in the 3′ UTR of mRNAs as epigenetic modifiers (39–41). It is elucidated that individual miRNAs can control the expression of more than one target mRNAs and that each mRNA may be regulated by multiple miRNAs (42). Over the past few decades, the studies that focused on the regulation of reproductive processes by miRNAs is gradually increasing. miRNAs-34/449 family members have been verified that play crucial roles in spermatogenesis, regulating spermatozoa maturation and functionality, the dysregulation of ciliogenesis in the efferent ductules is significantly impaired, leading to sperm aggregation and agglutination as well as to defective reabsorption of the seminiferous tubular fluids. Then the efferent ductules were obstructed and tubular fluids were accumulated, resulting in high hydrostatic pressure into the testis, leading to testicular dysfunction and spermatogenic failure (43). Besides, a previous study used the small RNA sequencing technology to reveal the differentially expressed miRNAs in High- and Low-motile sperm populations of cryopreserved semen and found that the DE miRNAs miR-17-5p, miR-26a-5p, miR-486-5p, miR-122-5p, miR-184, and miR-20a-5p may relate to reproductive processes (44). Thus, it can be seen that miRNAs could play great roles in regulating reproductive traits. Although there were systemically studies that related to the problem of male sterility of cattle-yak, the comprehensive exploration of comparing the miRNAs profiles with cattle-yak and their parents' generations were rarely can be seen.

In this study, we compared the DE miRNAs and DEGs among cattle, yak, and cattle-yak groups. In the H vs. P groups, we found a total of 47 up-regulated and 34 down-regulated miRNAs, and there were 7 known miRNAs (6 up-regulated and 1 down-regulated) that can be mapped to their targets. The target genes ESPL1, KDM6A, and BCORL1 may be engaged in reproductive processes. In many previous studies, ESPL1 (extra spindle pole bodies like 1) have been found involved in cellular mitosis and cell cycle process (45–47). KDM6A (lysine demethylase 6A) belongs to the KDM6 family of histone H3 lysine 27 (H3K27) demethylases, which is an *X-*linked protein that contains a catalytic Jumonji C (JmjC) domain can facilitate the removal of the methyl group on di- and trimethylated H3K27 (H3K27me2/3), it can not only manifest demethylase activity but also regulate gene expression and enhancer activation independently of its demethylase activity. Many studies had demonstrated the function of KDM6A in differentiation, development, regeneration, and tumor suppression (48–50). BCORL1 was also proved that can be a contributor to spermatogenesis (51). Furthermore, we merged the common differentially expressed miRNAs between the two comparison groups and found that the known miRNAs bta-miR-7 were down-regulated in cattle-yak. With the analysis of target genes, we found that the FANCA gene is associated with the processes of DNA damage repair and the variation of FANCA would induce premature ovarian insufficiency and further cause female fertility (52, 53). In this study, we quantified the expression levels of the FANCA gene in cattle, yak, and cattle-yak and detected higher relative expression levels in cattle-yak. We concluded that the FANCA overexpression would increase the DNA stability excessively and interfere with the processes of spermatocyte meiosis.

Furthermore, we performed GO, KEGG, and GSEA analyses to annotate the function of the DE miRNAs target genes and DEGs. With GO and KGEE enrichment annotation, we found that the up-regulated miRNAs target genes in cattle and yak were most engaged in negative regulation of ubiquitin-dependent protein catabolic process, positive regulation of cell migration, positive regulation of mitochondrial DNA replication, positive regulation of DNA demethylation, protein deubiquitination processes and cell cycle (bta04110), PI3K–Akt signaling pathway(bta04151), RNA polymerase (bta03020), Focal adhesion (bta04510), and relaxin signaling pathway (bta04926). In our previous study, we have described the function of lncRNA target genes preliminarily (38). In this work, we further used gene set enrichment analysis (GSEA) to expatiate the function of DE gene sets. We performed GSE GO and GSE KEGG to annotate the function of DEGs, respectively, and found that the DE gene sets were enriched in ATP binding, the developmental process in reproduction, DNA binding, transporter activity, extracellular region processes, MAPK signaling pathway, pathways in cancer, and focal adhesion pathways. Studies had proved that both noncatalytic ATP binding and stable ADP binding can mediate chemo-mechanical transduction in axonemal dynein (54). ATP levels were highly associated with the levels of GSK3α sperm hexokinase activity (55). As an efflux pump of the ABCG subfamily of the ATP-binding cassette (ABC) transporters, ATP-binding Cassette Transporter G2(ABCG2) was also detected in the blood-testis barrier and epididymal, which was proven to play a great role in epididymal sperm maturation (56–58). Mitogen-activated protein kinases (MAPKs) are important signal transducing enzymes that are involved in many facets of cellular regulation such as gene expression, cell proliferation, cell differentiation, programmed cell death, and cell motility (59). MAPK signaling pathway can regulate dynamics of tight junctions and adherens junctions, proliferation, and meiosis of germ cells, and proliferation and lactate production of Sertoli cells (60, 61). Focal adhesion kinase is a widely expressed protein-tyrosine that plays an important role in intracellular signal transduction pathways triggered in response to cell interactions with the extracellular matrix (62). Focal adhesion pathways of focal adhesion kinase-mediated have been implicated in a diverse array of cellular processes, such as cell migration, growth factor signaling, cell cycle progression, and cell survival (63). Such the processes and pathways in cattle and yak up-regulated target genes and gene sets may positively engage in the sperm cell proliferation and seminal tubules differentiation.

The spermatogenesis contains spermatocytogenesis (mitosis), meiosis, and spermiogenesis (64). With a cycle of several mitotic divisions, spermatogonia and primary spermatocytes were produced and stem cells were renewed. In the stage of meiosis, the genetic material was duplicated and exchanged and then four haploid round spermatids were produced. Lastly, the haploid spermatids were differentiated, and mature spermatozoa were released into the lumen of seminiferous tubules. However, the male hybrid of cattle and yak cannot finish the above processes. Histological examination of seminiferous tubules revealed that gonocytes and spermatocytes were established normally, however, spermatogenesis was arrested at the meiosis phase began 10 months after birth in the hybrids (65). With the testicular tissue histological characteristics, we found that the seminiferous tubules were highly vacuolated and there were few spermatids to be seen. Previous studies also demonstrated that although cattle-yak has the same number of chromosomes (2*n* = 60) as that of cattle and yak, most of the primary spermatocytes of cattle-yak performed morphologically abnormal and have no XY bivalents, which hindered the subsequent processes of meiosis and spermiogenesis (18). The genes that participated in meiosis such as MEI1, SYCP3, and DAZL have been detected significantly down-regulated in adult cattle-yak testes compared to those in yak testes (24, 66, 67). Besides, the target gene USP9X identified in this study is essential for proper spermatogenesis. USP9*X-*null spermatogenic cells underwent apoptotic cell death at the early spermatocyte stage and then caused subsequent aberrant spermiogenesis, which resulted in complete infertility of USP9X conditional knockout male mice (68). The comprehensive regulation of such miRNAs target genes and DE genes would disturb the spermatocyte's meiosis process and further influence the reproductive traits of cattle-yak.

## Materials and methods

### Animals and testis sample collection

Three male cattle (H1, H2, and H3), three male cattle-yak (P1, P2, and P3), and three male yaks (M1, M2, and M3) of good health and consistent diet levels, with the same growth conditions aged at 18 months were randomly selected on a pasture of Hongyuan country, Sichuan province of China. They were then divided into three groups (H, P, and M). Testicular tissue samples were collected and stored at −80°C until total RNA was extracted when these animals were slaughtered.

### Histological analysis of testicular tissues

Testicular tissues collected from nine samples were immersed in 4% phosphate-buffered formalin overnight. Then the fixed paraffin-embedded tissues were sectioned with 4μm thickness. The sections have further been stained with hematoxylin and eosin (HE) and toasted in an oven set to 70°C.

### RNA extraction, library preparation, and sequencing

Total RNA was extracted from nine testicular tissue samples with a Total RNA Extractor (Trizol) (Sangon Biotech, Shanghai, China) according to the manufacturer's instructions, then Qubit2.0 (Life Technologies, Thermo Fisher Scientific, USA) and agarose gel were used to detect the concentration and integrity of total RNA, respectively. After that, the RNA Mix with 3' Adapter was incubated 2 min at 70°C, and then T4 RNA Ligase 2(New England Biolabs, USA) was used to connect the 3' Adapter at 22°C for 1 h. Similarly, T4 RNA Ligase 1(New England Biolabs, USA) was used to connect the 5' Adapter at 20°C for 1 h. Then the connected product was used to reverse transcription with 5 × First Strand Buffer and PCR amplification (Table 5). The amplified cDNA library was used to detect the integrity with 12%-page gel, and was sequenced on the Illumina platform (Illumina Hiseq X Ten).

**Table 5:** The information on the primers used in qRT-PCR validation.

**Gene name**	**Sequence of primer (5'- 3')**	**Product length (bp)**	**Tm (°C)**
miR-449a	TGGCAGTGTATTGTTAGCTTGT		59.7
FANCA	F: AGTTTCCGATGCCGTTCA		
	R: CGTGTCTCCAGTTTACCTCC	110	54.7
MYRFL	F: TGGCAACCACATTGCCCTCCTT		
	R: TCCAACTGTCTTTGCATGGGGCT	133	60
SHF	F: CCGAACAGATGCCGAGAAC		
	R: ATGTGCATGAAACCCTGACTG	122	56
GAPDH	F: CTTCGGCATTGTGGAGGG		
	R: GGAGGCAGGGATGATGTTCT	130	61.3

### Quality control, reads mapping, and sequence analysis

FastQC was used for quality checks on raw data of nine samples. Cutadapt and trimmomatic (69) were then used to remove the adaptors and low-quality reads, respectively. Blastn was used to align to Rfam (70) database and then the reads that mapped to the bovine rRNA, sRNA, snRNA, and snoRNA were discarded. Bowtie (71) was used to map to the bovine reference genome(ARS-UCD1.3), exon, and intron sequence, the reads that can be mapped to the bovine genome and the intron region were retained. Novel miRNAs prediction and known miRNAs quantification were separately run with the miRDeep2 module and quantifier module of the miRDeep2 software (72).

### Differential expression analysis

The R package edgeR (73) was utilized to compare the expression level of miRNAs between H_vs_P and M_vs_P. Specifically, the lowly expressed miRNAs were filtered with the CPM normalization method, and the DE miRNAs among cattle, yak, and cattle-yak were screened with *P-*value < 0.05, |fold change| >2. We also carried on the Venn analysis to merge the common DE miRNAs between H_vs_P and M_vs_P for further analysis.

### Target prediction and function annotation

With the filtered DE miRNAs of H_vs_P and M_vs_P, miranda was used to predict their target genes, and then ClusterProfiler (74) was used to analyze their function with GO and KEGG enrichment. In our previous study, we have already preliminarily explored the function of the novel lncRNA target genes and differentially expressed genes (DEGs) (38). To further understand the DEGs expression patterns and their function, we utilized the gene set enrichment analysis (GSEA) to demo some meaningful processes and pathways.

### Net work analysis of DE miRNAs target genes

To understand the interactions among function processes and genes with pathways intuitively, igraph was used to draw the net plots and describe the correlations among pathways. Especially, the GO to GO, gene to pathways, and pathway to pathway plots were diagramed, respectively.

### qPCR validation and statistical analysis

To verify the accuracy of sequencing results, miRNAs and several target genes were randomly selected for qPCR validation. The identified DE miRNAs miR-449a and the target genes FANCA, MYRFL, and SHF of miR-7 were selected for further qRT-PCR experiment with specific primers and conditions (Tables 5, 6). The 2^−Δ*ΔCT*^ method was used to quantify the relative expression of the miRNAs and the target genes. Student's *t-*test with Graph Pad Prism 6 software was used to compare the differences among cattle, yak, and cattle-yak samples. ^*^p-value < 0.05 was considered statistically significant and ^**^p-value < 0.01 was considered as extremely statistically significant.

**Table 6:** PCR reaction system and conditions.

**Component**	**Volume (μl)**
RT-PCR product	17
Primer-F (10 μM)	4
Primer-R (with barcode, 10 μM)	4
KAPA	25
Total volume	50

95°C, 5 min → (94°C, 10 s → 55°C, 10 s → 72°C, 15 s)_12_ → 72°C, 5 min → 4°C, ∞_°_.

## Conclusions

In this study, we used high-throughput sequencing technology to explore miRNAs testicular tissue profiles of cattle, yaks, and cattle-yak. Finally, 47 u*p-*regulated miRNAs and 34 down-regulated miRNAs were identified in cattle between the comparison of cattle and cattle-yak; 16 u*p-*regulated and 21 down-regulated miRNAs were identified in yak samples between the comparison of yak and cattle-yak. Subsequently, we merged the same differentially expressed miRNAs between H vs. P and M vs. P. As a result, the known miRNA bta-miR-7 was commonly down-regulated in cattle-yak in the two comparison groups, revealing that miR-7 and its target genes MYRFL, FANCA, INSL3, and USP9X may play an extremely important role in reproductive processes. We also conducted GO, KEGG, and GSEA to annotate the function of differentially expressed genes and target candidates identified in nine samples, the results showed that the pathways and terms were associated with spermatogenesis, and reproduction processes. We also found that the membrane raft (GO:0045121) process, focal adhesion (bta04510) pathways, and ITGB3 gene were in the hub locations of the network. Our research is important to explore the molecular mechanism of cattle-yak male sterility and provide effective strategies to resolve it.

## Data availability statement

The datasets analyzed for this study have been deposited in the Genome Sequence Archive (Genomics, Proteomics & Bioinformatics 2021) in National Genomics Data Center (Nucleic Acids Res 2022), China National Center for Bioinformation / Beijing Institute of Genomics, Chinese Academy of Sciences (GSA: CRA006824) that are publicly accessible at https://ngdc.cncb.ac.cn/gsa (75, 76).

## Ethics statement

The animal study was reviewed and approved by Institutional Animal Care and Use Committee at the College of Animal Science and Technology, Sichuan Agricultural University (Permit Number: DKY2020050). Written informed consent was obtained from the owners for the participation of their animals in this study.

## Author contributions

SZ, WS, and XJ performed the data analysis and drafted the manuscript. SZ, XJ, and YL collected the testicular samples involved in this study. YL and S-YC contributed to laboratory experiments. SL and S-YC revised the manuscript. XJ and JW designed the study and also revised the manuscript. All authors read and approved the final manuscript.

## Funding

This research was funded by the National Key R&D Program of China (2021YFD1200403), the Sichuan innovation team of the national modern agricultural industry technology system (SCCXTD-2022-13), and the Key R&D Program of Sichuan Province (2021YFYZ0007).

## Conflict of interest

The authors declare that the research was conducted in the absence of any commercial or financial relationships that could be construed as a potential conflict of interest.

## Publisher's note

All claims expressed in this article are solely those of the authors and do not necessarily represent those of their affiliated organizations, or those of the publisher, the editors and the reviewers. Any product that may be evaluated in this article, or claim that may be made by its manufacturer, is not guaranteed or endorsed by the publisher.
