# Supplementary material for: The exploration of miRNAs and mRNA profiles revealed the molecular mechanisms of cattle-yak male infertility
Source: Front Vet Sci. 2022 Oct 5;9:974703. doi: 10.3389/fvets.2022.974703 (PMC9581192; doi:10.3389/fvets.2022.974703)
Supplement: Supplementary file 1 [file Data_Sheet_1.ZIP › supplementary materials/Supplementary table1.pdf]

Supplementary Table 1-1 DNA alignment results of nine samples

sample	database	Total reads	Total percentage	Unique reads	Unique percentage
h1	DNA	5448647	0.423845	1772587	0.528907
h2	DNA	6139838	0.476457	2086695	0.551548
h3	DNA	7122441	0.472622	2373291	0.546471
m1	DNA	4476534	0.418563	1954724	0.503643
m2	DNA	5736035	0.463151	2359750	0.53872
m3	DNA	6308983	0.425364	2757348	0.504082
p1	DNA	5515553	0.410284	2342296	0.504779
p2	DNA	6074855	0.40555	3236625	0.489107
p3	DNA	4997417	0.405315	1595278	0.499665

Supplementary Table 1-2 mRNA alignment results of nine samples

sample	database	Total reads	Total percentage	Unique reads	Unique percentage
h1	mRNA	1180891	0.0918604	718067	0.214258
h2	mRNA	1150048	0.0892447	758889	0.200587
h3	mRNA	1332317	0.0884081	853173	0.19645
m1	mRNA	1483045	0.138667	1061347	0.273461
m2	mRNA	1481642	0.119634	972171	0.221942
m3	mRNA	2021847	0.136317	1381740	0.252602
p1	mRNA	1812822	0.13485	1288731	0.27773
p2	mRNA	2438257	0.162775	1927520	0.29128
p3	mRNA	1348550	0.109374	789809	0.24738

Supplementary Table 1-3 rRNA database alignment results of nine samples

sample	database	Total reads	Total percentage	Unique reads	Unique percentage
h1	rRNA	471859	0.036706	66587	0.0198683
h2	rRNA	594721	0.046151	73325	0.019381
h3	rRNA	774140	0.051369	87468	0.0201403
m1	rRNA	910813	0.085163	121315	0.0312573
m2	rRNA	1059582	0.085555	123987	0.0283056
m3	rRNA	1131794	0.076308	108082	0.0197589
p1	rRNA	1155242	0.085935	122028	0.0262978
p2	rRNA	945527	0.063122	118346	0.017884
p3	rRNA	1016321	0.082429	93815	0.0293842

Supplementary Table 1-4 snoRNA database alignment results of nine samples

sample	database	Total reads	Total percentage	Unique reads	Unique percentage
h1	snoRNA	106474	0.00828251	27144	0.00809927
h2	snoRNA	100594	0.00780618	28202	0.00745425
h3	snoRNA	120800	0.00801589	31560	0.00726696
m1	snoRNA	258541	0.024174	48600	0.012522
m2	snoRNA	229444	0.0185262	44139	0.0100767
m3	snoRNA	432660	0.0291708	64718	0.0118314
p1	snoRNA	418723	0.0311475	57003	0.0122845
p2	snoRNA	293366	0.0195848	56103	0.00847807
p3	snoRNA	241766	0.0196084	41954	0.0131406

Supplementary Table 1-5 snRNA database alignment results of nine samples

sample	database	Total reads	Total percentage	Unique reads	Unique percentage
h1	snRNA	77167	0.006003	22186	0.00661989
h2	snRNA	89200	0.006922	24121	0.00637558
h3	snRNA	108666	0.007211	27880	0.00641961
m1	snRNA	116495	0.010893	27393	0.00705793
m2	snRNA	137918	0.011136	31033	0.00708468
m3	snRNA	177411	0.011961	36308	0.00663761
p1	snRNA	170384	0.012674	32861	0.00708175
p2	snRNA	182917	0.012211	38653	0.0058411
p3	snRNA	117768	0.009552	23382	0.00732359

Supplementary Table 1-6 tRNA database alignment results of nine samples

sample	database	Total reads	Total percentage	Unique reads	Unique percentage
h1	tRNA	3773380	0.293528	68770	0.0205197
h2	tRNA	2067769	0.160461	67285	0.0177845
h3	tRNA	2268746	0.150546	74596	0.0171764
m1	tRNA	2713315	0.253699	77728	0.020027
m2	tRNA	1991640	0.160813	78976	0.0180298
m3	tRNA	3347190	0.225674	91529	0.0167328
p1	tRNA	3348763	0.249104	94716	0.0204119
p2	tRNA	3523024	0.235193	93970	0.0142004
p3	tRNA	3751280	0.304247	86937	0.02723
