# Supplementary material for: The exploration of miRNAs and mRNA profiles revealed the molecular mechanisms of cattle-yak male infertility
Source: Front Vet Sci. 2022 Oct 5;9:974703. doi: 10.3389/fvets.2022.974703 (PMC9581192; doi:10.3389/fvets.2022.974703)
Supplement: Supplementary file 1 [file Data_Sheet_1.ZIP › supplementary materials/Supplementary table2.pdf]

Supplementary Table 2-1 The differentially expressed miRNAs between cattle(H) and cattle-yak(P).

id	log2FoldChange	log2CPM	p-value	p-adj	regulated
bta-novel-120-mature	8.430884	4.588426	1.77E-13	1.03E-10	up
bta-novel-110-mature	8.262681	4.431661	6.59E-11	2.56E-08	up
bta-novel-154-mature	7.458468	3.662599	8.35E-09	1.95E-06	up
bta-novel-114-mature	7.060919	3.305166	5.67E-08	9.00E-06	up
bta-miR-34b	5.430329	9.951872	0.000670009	0.033937423	up
bta-novel-173-mature	5.301668	3.777943	1.54E-05	0.00119748	up
bta-novel-142-mature	4.831273	1.857784	0.018319165	0.346266409	up
bta-miR-34c	4.564578	15.33435	0.002587206	0.094190455	up
bta-miR-449b	4.432023	7.763628	0.003401305	0.116178018	up
bta-miR-449a	4.395882	12.17588	0.021708935	0.395170455	up
bta-novel-172-mature	4.043426	2.299045	0.00042812	0.02493797	up
bta-novel-128-mature	3.943484	2.269172	0.012041381	0.275062919	up
bta-novel-148-mature	3.921308	2.232301	0.014119626	0.304617851	up
bta-novel-18-star	3.698384	3.404809	1.80E-07	2.10E-05	up
bta-novel-185-mature	3.530816	2.037467	0.003072175	0.108457081	up
bta-miR-190b	3.474138	4.400322	8.39E-06	0.000751975	up
bta-novel-163-mature	3.438074	8.678416	2.02E-06	0.00019632	up
bta-novel-174-mature	3.272684	2.676691	0.001170008	0.05242534	up
bta-novel-24-mature	3.16267	5.117273	0.013110352	0.293722309	up
bta-miR-146a	3.148904	13.93316	1.02E-13	1.03E-10	up
bta-miR-449c	3.035521	4.220758	0.016975293	0.346266409	up
bta-novel-149-mature	2.98055	2.827241	0.005576748	0.175592211	up
bta-novel-139-mature	2.812304	2.644452	0.001360132	0.05463979	up
bta-novel-147-mature	2.812304	2.644452	0.001360132	0.05463979	up
bta-miR-2285b	2.769263	3.882896	4.13E-05	0.002832297	up
bta-miR-2425-5p	2.746397	2.108955	0.033795875	0.49215243	up
bta-novel-125-mature	2.726319	3.707195	0.022546936	0.397987579	up
bta-novel-42-mature	2.501396	2.532192	0.024089475	0.406728084	up
bta-novel-62-mature	2.484285	2.806559	0.025169478	0.412992134	up
bta-miR-95	2.44341	4.26958	0.029293916	0.461181246	up
bta-miR-122	2.316013	3.615935	0.043242554	0.592677352	up
bta-novel-6-mature	2.260296	3.069769	0.000728176	0.034784679	up
bta-miR-2285h	2.216068	3.094191	0.002517945	0.094190455	up
bta-miR-9-3p	2.117166	5.28466	0.004551117	0.147279188	up
bta-novel-1-star	2.113663	6.68486	0.008465686	0.228239168	up
bta-miR-335	2.08474	11.44975	4.12E-07	4.37E-05	up
bta-miR-184	1.95091	5.140499	0.005975201	0.183187096	up
bta-miR-2336	1.943145	3.272005	0.008707756	0.228239168	up
bta-miR-18a	1.834719	7.103782	0.00066469	0.033937423	up
bta-miR-2284x	1.784195	6.124088	1.48E-05	0.00119748	up
bta-miR-7	1.783929	9.525128	0.00025328	0.015530066	up

bta-miR-2284y	1.776036	6.134096	1.66E-05	0.001210858	up
bta-miR-6123	1.644458	5.914324	0.006898235	0.196010814	up
bta-miR-9-5p	1.501853	7.694732	0.006708054	0.196010814	up
bta-miR-93	1.044483	11.55757	0.018427912	0.346266409	up
bta-miR-19a	1.039544	8.146738	0.010320746	0.254807956	up
bta-miR-296-3p	1.015611	6.48256	0.033150663	0.49215243	up
bta-miR-411a	-1.1249	8.75017	0.03836317	0.545037725	down
bta-miR-455-3p	-1.13228	8.069027	0.023580176	0.406728084	down
bta-miR-708	-1.29603	9.536147	0.006867384	0.196010814	down
bta-miR-574	-1.446	7.168405	0.000746452	0.034784679	down
bta-novel-79-mature	-1.47224	12.06273	0.00061586	0.033937423	down
bta-miR-1343-3p	-1.47239	6.080659	0.011495957	0.273322253	down
bta-miR-2285u	-1.48184	5.306928	0.033626601	0.49215243	down
bta-miR-424-5p	-1.48206	11.54815	0.000237693	0.015384012	down
bta-novel-164-mature	-1.48464	4.170994	0.011978803	0.275062919	down
bta-miR-2285o	-1.504	5.280979	0.03246604	0.49215243	down
bta-novel-1-mature	-1.55415	14.59167	0.046802654	0.612641479	down
bta-novel-115-mature	-1.55415	14.59167	0.046802654	0.612641479	down
bta-novel-182-mature	-1.74339	5.43946	0.015396741	0.320307194	down
bta-novel-12-mature	-1.81073	11.09212	0.019741549	0.365061978	down
bta-novel-150-mature	-2.43339	5.98306	0.003490327	0.116178018	down
bta-miR-2285i	-2.44643	2.882179	0.017622427	0.346266409	down
bta-miR-33a	-2.51812	4.025868	0.001343177	0.05463979	down
bta-novel-103-mature	-2.54369	2.91705	0.008816105	0.228239168	down
bta-novel-170-mature	-3.41983	2.858361	0.010498525	0.254807956	down
bta-novel-8-mature	-4.12421	1.872575	0.046042366	0.612641479	down
bta-novel-124-mature	-4.13156	1.875413	0.044441488	0.602027137	down
bta-novel-156-mature	-4.15948	1.892865	0.041587694	0.583730887	down
bta-novel-167-mature	-4.29361	1.936721	0.048306949	0.625306618	down
bta-novel-130-mature	-4.55198	2.054479	0.030586657	0.475112746	down
bta-novel-145-mature	-4.56717	6.029916	0.001895341	0.073602406	down
bta-novel-51-mature	-4.66836	2.112723	0.01726781	0.346266409	down
bta-novel-50-mature	-4.68232	2.117882	0.017882647	0.346266409	down
bta-novel-85-mature	-5.32777	2.510707	0.04259131	0.590700902	down
bta-novel-33-mature	-5.42757	2.562245	0.007146764	0.198237607	down
bta-miR-2284j	-5.63393	2.687397	0.009667569	0.244841685	down
bta-novel-146-mature	-7.27575	3.842709	6.18E-08	9.00E-06	down
bta-novel-21-mature	-7.34397	3.897527	2.05E-08	3.99E-06	down
bta-novel-152-mature	-7.64674	4.149401	1.16E-07	1.51E-05	down
bta-novel-123-mature	-9.34248	5.619578	1.75E-10	5.08E-08	down

Supplementary Table 2-2 The differentially expressed miRNAs between yak(M) and cattle-yak(P).

id	log2FoldChange	log2CPM	pvalue	padj	regulated
bta-miR-451	3.871109822	2.755158908	0.001571602	0.366183259	up
bta-novel-173-mature	3.323572809	2.437120778	0.013925833	0.951312298	up
bta-novel-151-mature	3.074852765	2.631465413	0.004204031	0.544188462	up
bta-novel-25-mature	2.187005643	3.135884339	0.014698387	0.951312298	up
bta-novel-98-mature	2.187005643	3.135884339	0.014698387	0.951312298	up
bta-novel-63-mature	1.995368309	2.740296079	0.026412317	1	up
bta-miR-2318	1.951073635	2.466424066	0.01795185	1	up
bta-miR-144	1.913245423	4.040026356	0.007224231	0.810909297	up
bta-novel-36-mature	1.880957974	2.211042128	0.037769139	1	up
bta-miR-2285j	1.705669047	3.034329905	0.012614428	0.951312298	up
bta-miR-2285b	1.639361049	3.29739543	0.008926306	0.866595574	up
bta-miR-1197	1.325811195	2.954887136	0.041514665	1	up
bta-miR-154b	1.173215028	3.817347228	0.04476235	1	up
bta-miR-502a	1.067378738	6.660336508	0.023277273	1	up
bta-miR-31	1.027780311	10.94021882	0.046891107	1	up
bta-miR-7	1.00590343	9.10135733	0.042882475	1	up
bta-miR-34a	-1.027263476	6.63873076	0.039484911	1	down
bta-novel-8-star	-1.032376594	5.275679978	0.020362214	1	down
bta-novel-9-mature	-1.032376594	5.275679978	0.020362214	1	down
bta-miR-193b	-1.058936825	7.183022444	0.007656654	0.810909297	down
bta-novel-163-mature	-1.324211452	5.832219226	0.025183332	1	down
bta-novel-79-mature	-1.414056731	12.2409307	0.002903146	0.422770641	down
bta-novel-152-mature	-1.476215978	4.539071777	0.014093282	0.951312298	down
bta-miR-6526	-1.560696961	12.10659103	0.018379845	1	down
bta-novel-11-star	-1.609087748	3.028761757	0.049542384	1	down
bta-novel-143-mature	-1.757413471	3.143658666	0.039311975	1	down
bta-novel-164-mature	-2.047633885	4.099395475	0.0001905	0.199247495	down
bta-novel-150-mature	-2.143744267	6.143532087	0.034224288	1	down
bta-novel-45-mature	-2.352059119	1.797475899	0.043659324	1	down
bta-novel-115-star	-2.713524435	8.645648124	0.002369561	0.409033558	down
bta-novel-12-mature	-2.934660952	11.11061451	0.000735773	0.214293979	down
bta-novel-11-mature	-3.071297487	4.654142248	0.002457712	0.409033558	down
bta-novel-1-mature	-3.147717034	14.52690285	0.000513084	0.199247495	down
bta-novel-115-mature	-3.147717034	14.52690285	0.000513084	0.199247495	down
bta-novel-130-mature	-3.253280196	1.816468164	0.034318961	1	down
bta-miR-196b	-3.977727377	3.704238422	0.031813582	1	down
bta-novel-167-mature	-4.81952285	1.64307922	0.018932623	1	down
